# Fire risk assessment of high-rise buildings under construction based on unascertained measure theory

**DOI:** 10.1371/journal.pone.0239166

**Published:** 2020-09-14

**Authors:** Wenlong Li, Huimin Li, Yijun Liu, Sunmeng Wang, Xingwang Pei, Qian Li

**Affiliations:** 1 School of Civil Engineering, Xi’an University of Architecture and Technology, Xi’an, Shaanxi, China; 2 School of Management, Xi’an University of Architecture and Technology, Xi’an, Shaanxi, China; Shandong University of Science and Technology, CHINA

## Abstract

To prevent fire accidents in high-rise buildings under construction, in this paper, the fire risk assessment of such buildings is studied. First, based on project investigation and a literature review, a fire risk assessment index system suitable for high-rise buildings under construction was established. Second, the unascertained measure theory was applied to establish a fire risk assessment model for high-rise buildings under construction. The index weight was determined by the entropy weight method. Finally, taking a high-rise building project in Xi’an, China, as an example, the feasibility and rationality of the fire risk assessment index system and assessment model were verified. This research provides a new method for objectively assessing the fire risk of high-rise buildings under construction and provides a certain reference for controlling the fire risk of high-rise buildings under construction.

## Introduction

The continuous progress of society and the development of the economy have resulted in gradual urbanization and the rapid construction of various types of buildings [1, 2]. At present, the engineering projects under construction can be seen everywhere. Due to the large amount of combustible and flammable materials on the construction site, many fire and heat sources, poor fire fighting conditions, and high fire risk, and once a fire occurs, it is difficult to fight the fire. The frequent fires of engineering projects under construction have caused serious safety accidents, caused serious property losses and casualties, and brought extremely bad social impacts [3]. At the same time, it will also affect the sustainable development of society and the environment [4]. Therefore, the fire risk of engineering projects under construction has attracted the attention of all sectors of society.

The fire risks of high-rise building under construction are relatively large, with many influencing factors and thus high complexity and uncertainty, mainly in the following aspects. On the one hand, high-rise buildings under construction require a large amount of work, a long construction period, and a complex and variable construction environment, and these factors increase fire risk uncertainty. Especially during the peak period of construction, there are many mixed operations, open-fire operations, and fire hazards, which can easily lead to fire accidents. On the other hand, the high-rise buildings under construction are still in the construction state, most of the fire control systems of the buildings have not been installed in place, or are not in use even though they have been installed. Instead, there are only some temporary fire control facilities on the construction site, and the fire water source is limited. Therefore, in the event of a fire, the fire spreads quickly and is difficult to extinguish.

Therefore, it is important to understand the hidden fire hazards present in high-rise buildings under construction, to perform scientific risk assessments and to take effective preventive measures.

## Literature review

The literature review and analysis are divided into two parts.

### Building fire risk assessment

To reduce the probability of building fires and to prevent and control such fires, much research has been done on building fire risk. In the 1980s, with the continuous progress of science and technology, ultrahigh buildings and super-large buildings began to appear; however, the current design specifications could not meet the fire protection requirements of the new buildings. Therefore, performance-based fire protection design was proposed. In 1985, the UK promulgated the first performance-based fire protection code. Thereafter, some developed countries researched performance-based fire protection design and related fire safety engineering theories and technologies and explored performance-based fire protection design. From 1996 to 2002, four international symposia on performance-based design norms and design methods were held, showing that performance-based fire protection design had become an international trend. Since then, an increasing number of experts and scholars have researched building fire risk. Kang et al. calculated the fire safety assessment level of high-rise buildings by using fuzzy centralization theory, and the developed method was both applicable and practical [5]. Ding et al. developed a smart fire risk estimation model for high-rising buildings by using the back propagation (BP) neural network [6]. Ren designed a model for assessing the fire risks of logistics warehouses by using the analytic hierarchy process (AHP) method [7]. Xin et al. analyzed a large number of fire incidents in China, determined the characteristics and main factors of the fires, and evaluated the risk levels of residential buildings [8]. Wu et al. presented a diagnostic assessment of fire safety by using the extension engineering method, which can be applied for all kinds of buildings [9]. Gao et al. established a fuzzy analytic hierarchy process model that aimed to assess the tunnel fire risk of subways by combining the fuzzy consistent matrix with AHP [10]. Chen et al. established a building fire risk assessment system for factories, hotels, malls, schools and public buildings and applied AHP to evaluate building fire risk [11]. Sun et al. introduced fuzzy mathematics into the analytic hierarchy process; furthermore, the analytic hierarchy process-fuzzy comprehensive evaluation (AHP-FCE) method was used to evaluate risk, and the traditional quantitative evaluation method was integrated with the qualitative evaluation method [12]. Roshan et al. assessed fire risk and economic loss by using event tree analysis [13]. Lau et al. proposed a fire risk scoring system and applied it for determine the fire risk of residential buildings [14]. Wei et al. established a fire risk assessment model based on support vector machine (SVM) theory, and the method was precise even when given a small number of samples [15]. Wei et al. proposed a fast fire risk assessment method based on fuzzy mathematics and the SVM algorithm [16]. Liu et al. proposed a fire risk assessment system for large-scale commercial buildings by using the structure entropy weight method [17]. Li et al. established a mathematical model by using the gray risk degree method, the analytic hierarchy process and the fuzzy evaluation method [18]. Bart et al. developed a quantitative risk assessment method that can quantify the fire safety level through the failure probability, individual risk and social risk [19]. Sun et al. expounded the procedures and methods of fire risk assessment for super-high-rise buildings and quantified the possibility and consequences of a fire [20]. Qian et al. established an urban fire risk assessment index system according to the possibility and severity of the fire and constructed an evaluation model based on regression with latent variables [21]. Hassanain et al. developed a fire safety evaluation tool that can evaluate existing restaurant facilities to identify and eliminate fire hazards [22]. Omidvari et al. proposed a model based on the analytical hierarchy process and failure mode and effect analysis logic [23]. Li et al. built a fire risk assessment system for coal mines based on the TOPSIS method [24]. Zheng conducted a fire risk assessment of the stadium used for the National Games based on the AHP method to ensure safety [25].

Many researchers have introduced and systematically summarized models and methods for quantitatively assessing building fire risk [26]. In addition, there are many fire risk assessment methods [27, 28] (e.g., expert scoring, Delphi, analytic hierarchy process, fuzzy comprehensive evaluation, fault tree analysis, grey comprehensive evaluation, SVM, TOPSIS, artificial neural network, matter element extension). Researchers have applied these methods for fire risk assessment.

In summary, there are many studies on fire risk, and good applications have been developed. Relevant research is mainly focused on the fire risk of existing buildings. There is relatively little research on the fire risk of buildings that are under construction and even less research on the fire risks of high-rise buildings under construction. Therefore, in this paper, high-rise buildings under construction are considered as the research object, the characteristics of fire accidents in the high-rise building under construction are summarized, a fire risk assessment is conducted for high-rise buildings under construction.

However, the fire risks of high-rise buildings under construction are relatively large, with many influencing factors and thus high complexity and uncertainty. In addition, there are many uncertainties associated with the assessment index and the assessment process. Therefore, reasonable mathematical methods are needed for fire risk assessment of high-rise buildings under construction.

### Unascertained measure theory

Unascertained information and its mathematical processing theory were first proposed by Wang Guangyuan in 1990 [29]. Unlike fuzzy information, random information and gray information, unascertained information indicates that people do not fully grasp the real quantitative relationships or states being considered, which causes subjective and cognitive uncertainty in the minds of decision makers and evaluators. It can be said that all systems with behavioral factors are unascertained. To develop a method for quantitatively describing the unascertained state or the unascertained size of something, Liu Kaidi et al. [30] established unascertained mathematical theory and proposed an evaluation model for unascertained measure theory to describe an unascertained state or an unascertained nature by using a real number in [0,1].

Thereafter, unascertained measure theory has been rapidly developed and widely applied in many fields, such as mining risk assessment [31, 32], geotechnical risk evaluation [33, 34], pipeline risk assessment [35], geological risk assessment [36], ecological risk assessment [37], chemical safety evaluation [38], and social evaluation [39]. According to the above description, unascertained measure theory can effectively and quantitatively analyze various uncertain factors. Furthermore, it can avoid the incompleteness of risk assessment indexes due to the uncertainty of the influencing factors, and it can avoid the shortcomings of the subjectivity of risk assessment results caused by expert scoring.

This paper applies unascertained measure theory to the fire risk assessment of high-rise buildings under construction to address many uncertainties of fire risk assessment. It is hoped that the model can provide a new idea for fire risk assessment of high-rise buildings under construction.

## Model development

**Fig 1:**
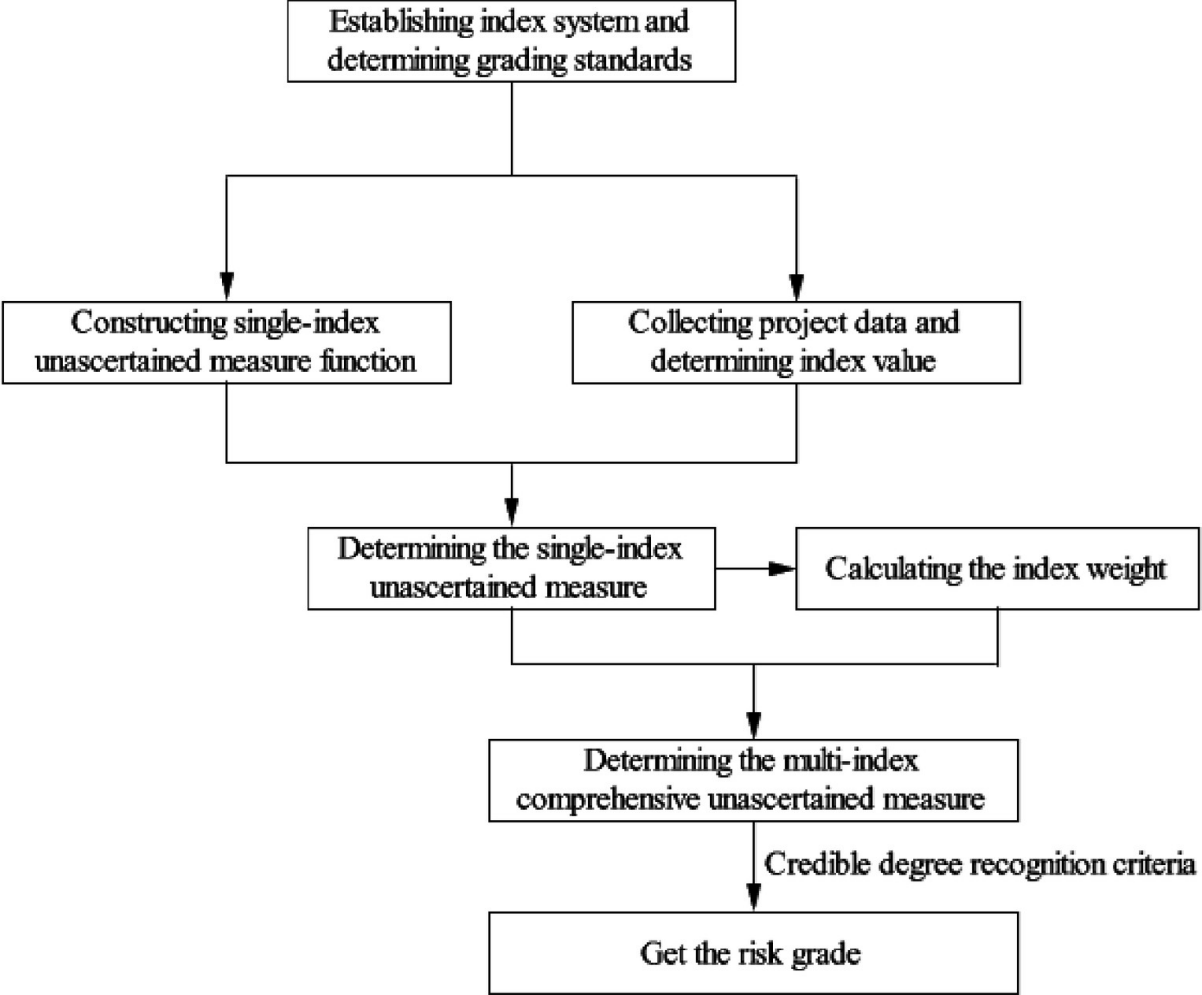
The algorithm for this study.

The purpose of this study is to conduct a fire risk assessment of high-rise buildings under construction. The algorithm used in this study is illustrated in Fig 1.

### Fire risk assessment index system and index grading ranges

#### Establishment of the fire risk assessment index system

Establishing a scientific and comprehensive assessment index system is the key to risk assessment, as it affects the reliability and accuracy of the assessment results. For high-rise buildings, the chimney effect can occur, which greatly impacts fire risk. Furthermore, for high-rise buildings under construction, the site is complex and changing, and various fire influencing factors are uncertain. Therefore, the establishment of the assessment index system is very difficult.

Based on the above literature review in the “Building fire risk assessment” and relevant standards [40–43], visited and consulted the owner, supervisory unit, design unit, fire brigade and other related units, the index system and index grading standards were determined. A fire risk assessment index system that is suitable for high-rise buildings under construction is established as shown in Table 1.

**Table 1:** Fire risk assessment index system for high-rise buildings under construction.

First-level index	Second-level index	Third-level index
Passive fire prevention factor (V_1_)	Architectural design (V_11_)	Fireproof endurance rating of building material (V_111_)
		Fire and smoke prevention zoning (V_112_)
		Fire separation distance (V_113_)
		Building height (V_114_)
	Fire load (V_12_)	Fire load density (V_121_)
Active fire prevention factor (V_2_)	Building fire extinguishing facilities (V_21_)	Allocation situation of temporary fire fighting equipment (V_211_)
		Fire detection and automatic alarm (V_212_)
		Temporary fire water-supply system (V_213_)
	Fire-fighting capacity of fire brigade (V_22_)	Fire lane (V_221_)
		Firefighter business level (V_222_)
Safe evacuation (V_3_)	Safe evacuation channel (V_31_)	Evacuation channel condition (V_311_)
		Setup situation of safety export (V_312_)
		Safe evacuation route and distance (V_313_)
	Safe evacuation facilities (V_32_)	Safety evacuation indication sign (V_321_)
		Emergency lighting (V_322_)
	Site personnel situation (V_33_)	Pedestrian flow situation of construction site (V_331_)
		Fire awareness of on-site personnel (V_332_)
	Emergency plan (V_34_)	Emergency relief material (V_341_)
		Preparation of emergency rescue plan (V_342_)
		Drill of emergency rescue plan (V_343_)
Fire safety management (V_4_)	Establishment of management system (V_41_)	Establishment and implementation of fire control system (V_411_)
		Division of safety duty (V_412_)
	Daily fire management work (V_42_)	Maintenance of fire fighting facility (V_421_)
		Regular inspection of fire safety (V_422_)
		Education and training of fire safety (V_423_)
		Management of flammable and explosive material (V_424_)
	Managerial personnel factor (V_43_)	Mastery situation of fire control knowledge and skill (V_431_)
		Managerial personnel business level (V_432_)
Fire hazard source control (V_5_)	Human factor (V_51_)	Production hot work (V_511_)
		Careless use of electricity (V_512_)
		Careless smoking (V_512_)
	Material factor (V_52_)	Material stacking condition (V_521_)
		Electrical equipment condition (V_522_)

#### Determination of the index grading standards

This paper determines the index grading standards through theoretical research and expert experience and converts qualitative indexes into semiquantitative indexes. Based on the above literature review, relevant standards [40–43], project investigation and consulting expert opinions, the fire risk assessment is divided into five grades, i.e., Grade I, Grade II, Grade III, Grade IV and Grade V, which refer to very low, low, moderate, high, very high fire risks, respectively. The specific index grading standards are shown in Table 2.

**Table 2:** Index grading standards.

Index	Grade I (*C*_1_)	Grade II (*C*_2_)	Grade III (*C*_3_)	Grade IV (*C*_4_)	Grade V (*C*_5_)
	(90, 100]	(80, 90]	(70, 80]	(60, 70]	[0, 60]
V_111_	<1%	1%-5%	5%-10%	10%-15%	≥15%
V_112_	<1%	1%-5%	5%-10%	10%-15%	≥15%
V_113_	<1%	1%-5%	5%-10%	10%-15%	≥15%
V_114_	Below 3 floors	4 to 6 floors	7 to 9 floors	10 floors to the top	Capping and Decoration Stage
V_121_	≤200	200–400	400–600	600–800	>800
V_211_	<1%	1%-5%	5%-10%	10%-15%	≥15%
V_212_	Very good usability and very high safety	Good usability and high safety	General usability and general safety	Poor usability and low safety	Not usable and very low safety
V_213_	<1%	1%-5%	5%-10%	10%-15%	≥15%
V_221_	<1%	1%-5%	5%-10%	10%-15%	≥15%
V_222_	Very high level of fire fighting	High level of fire fighting	General level of fire fighting	Poor level of fire fighting	Very poor level of fire fighting
V_311_	<1%	1%-5%	5%-10%	10%-15%	≥15%
V_312_	<1%	1%-5%	5%-10%	10%-15%	≥15%
V_313_	<1%	1%-5%	5%-10%	10%-15%	≥15%
V_321_	<1%	1%-5%	5%-10%	10%-15%	≥15%
V_322_	<1%	1%-5%	5%-10%	10%-15%	≥15%
V_331_	<0.5	0.5–1	1–1.5	1.5–2	2–2.5
V_332_	Very high fire awareness	High fire awareness	General fire awareness	Low fire awareness	Very low fire awareness
V_341_	Very adequate	Adequate	General	Inadequate	Very inadequate
V_342_	Compliant	Basically compliant	Generally compliant	Less compliant	Incompliant
V_343_	Regular	General	Occasional	Few	Never
V_411_	Very good	Good	General	Bad	Very bad
V_412_	Very clear and reasonable	Basically clear and reasonable	Generally clear and reasonable	Less clear and reasonable	Not clear and reasonable
V_421_	Very good	Good	General	Bad	Very bad
V_422_	Regular	General	Occasional	Few	Never
V_423_	Regular	General	Occasional	Few	Never
V_424_	Very safe and reasonable	Basically safe and reasonable	Generally safe and reasonable	Less safe and reasonable	Not safe and reasonable
V_431_	Very skilled	Basically skilled	Generally skilled	Less skilled	Very unskilled
V_432_	Very strong	Strong	General	Weak	Very weak
V_511_	The operation is strictly in conformity with the specifications and very high safety	The operation is in conformity with the specifications and high safety	The operation is general in conformity with the specifications and general safety	The operation is not in conformity with the specifications and low safety	The operation is strictly not in conformity with the specifications and very low safety
V_512_	The operation is strictly in conformity with the specifications and very high safety	The operation is in conformity with the specifications and high safety	The operation is general in conformity with the specifications and general safety	The operation is not in conformity with the specifications and low safety	The operation is strictly not in conformity with the specifications and very low safety
V_512_	Compliant	Basically compliant	Generally compliant	Less compliant	Incompliant
V_521_	The stacking is strictly in conformity with the specifications and very high safety	The stacking is in conformity with the specifications and high safety	The stacking is general in conformity with the specifications and general safety	The stacking is not in conformity with the specifications and low safety	The stacking is strictly not in conformity with the specifications and very low safety
V_522_	The erection and utilization are strictly in conformity with the specifications and very high safety	The erection and utilization are in conformity with the specifications and high safety	The erection and utilization are general in conformity with the specifications and general safety	The erection and utilization are not in conformity with the specifications and low safety	The erection and utilization are strictly not in conformity with the specifications and very low safety

The index grading standards of V_111_, V_112_, V_113_, V_211_, V_213_, V_311_, V_312_, V_313_, V_321_, and V_322_ are expressed by the ratio of the number that does not meet the requirements of the specification to the total number, while the index grading standard V_331_ is expressed by the ratio of the number of people in the peak period to the total building area.

### Unascertained measure theory

Suppose that the assessment object *X* = {*X*_1_,*X*_2_,⋯,*X*_*n*_} and the assessment index set *V* = {*V*_1_,*V*_2_,⋯,*V*_*m*_}. If *x*_*ij*_ denotes the measured value of the i-th assessment object *X*_*i*_ with respect to the j-th assessment index *V*_*j*_, then *X*_*i*_ can be expressed as an m-dimensional vector {*x*_*i*1_,*x*_*i*2_,⋯,*x*_*im*_}. Suppose that the assessment grade space *C* = {*C*_1_,*C*_2_,⋯,*C*_*p*_}, where *C*_*k*_(*k* = 1,2,⋯,*p*) is the k-th assessment grade, and suppose that the k-th grade is higher than the *k*+1-th grade in the risk assessment process, i.e., *C*_*k*_>*C*_*k*+1_. If *C*_1_>*C*_2_>⋯>*C*_*p*_ or *C*_1_<*C*_2_<⋯<*C*_*p*_ is satisfied, then {*C*_1_,*C*_2_,⋯,*C*_*p*_} is an ordered segmentation class of assessment space *C*.

#### Single-index unascertained measure

If *μ*_*ijk*_ = *μ*(*x*_*ij*_∈*C*_*k*_) denotes the degree to which the measured value *x*_*ij*_ belongs to the *k*-th assessment grade *C*_*k*_, then

$$0\leq \mu \left(x_{ij}\in C_{k}\right)\leq 1\;(i=1,2,\cdots ,n;j=1,2,\cdots ,m;k=1,2,\cdots ,p)$$ (1)

$$\mu \left(x_{ij}\in C\right)=1(i=1,2,\cdots ,n;j=1,2,\cdots ,m)$$ (2)

$$\mu \left|x_{ij}\in \overset{k}{\underset{l=1}{\cup }}C_{l}\right|=\sum_{l=1}^{k}\mu \left(x_{ij}\in C_{l}\right)(k=1,2,\cdots ,p)$$ (3)

Eq (1) is called nonnegative boundedness, Eq (2) is called normalization, and Eq (3) is called additivity. If *μ* satisfies Eqs (1)–(3), then *μ* is called the unascertained measure, which is abbreviated as measure.

For every assessment object *X*_*i*_(*i* = 1,2,⋯,*n*), the matrix of (*μ*_*ijk*_)_*m*×*p*_ is called the single-index unascertained measure matrix of *X*_*i*_, as shown in Eq (4).

$${\left(\mu_{ijk}\right)}_{m\times p}=\left[\begin{array}{cccc} \mu_{i11} & \mu_{i12} & \cdots & \mu_{i1p}\\ \mu_{i21} & \mu_{i22} & \cdots & \mu_{i2p}\\ \vdots & \vdots & \ddots & \vdots \\ \mu_{im1} & \mu_{im2} & \cdots & \mu_{imp} \end{array}\right]$$ (4)

Before establishing the single-index unascertained measure matrix, it is necessary to establish a single-index unascertained measure function. At present, the construction methods of a single-index unascertained measure function mainly include linear, exponential, parabolic and sinusoidal methods [44]. Regardless of the type of simulation function used, it must satisfy the limiting conditions of Eqs (1)–(3). To operate simply and easily, this paper adopted the linear unascertained measure function, and the calculation expression is as follows [45]:

$$\{\begin{array}{c} \mu_{i}(x)=\{\begin{array}{cc} \frac{-x}{a_{i+1}-a_{i}}+\frac{a_{i+1}}{a_{i+1}-a_{i}} & a_{i}<x\leq a_{i+1}\\ 0 & x>a_{i+1} \end{array}\\ \mu_{i+1}(x)=\{\begin{array}{cc} 0 & x\leq a_{i}\\ \frac{x}{a_{i+1}-a_{i}}-\frac{a_{i}}{a_{i+1}-a_{i}} & a_{i}<x\leq a_{i+1} \end{array} \end{array}$$ (5)

#### Multi-index comprehensive unascertained measure

Given that *μ*_*ik*_ = *μ*(*X*_*i*_∈*C*_*k*_) denotes the degree to which the assessment object *X*_*i*_ belongs to the k-th assessment grade *C*_*k*_, as shown in Eq (6), where 0≤*μ*_*ik*_≤1 and $\sum_{k=1}^{p}\mu_{ik}=1$ are satisfied, the vector {*μ*_*i*1_,*μ*_*i*2_,⋯,*μ*_*ip*_} is called the multi-index comprehensive unascertained measure vector of *X*_*i*_.

$$\mu_{ik}=\sum_{j=1}^{m}w_{j}\cdot \mu_{ijk}(i=1,2,\cdots ,n;k=1,2,\cdots ,p)$$ (6)

where *w*_*j*_ is the index weight. The specific index weight calculation process is detailed in the following section.

#### Credible degree recognition

To get the final assessment result, credible degree recognition criteria are introduced. Supposed that *λ* (*λ*≥0.5; usually, *λ* = 0.6 or 0.7) is the credible degree, if *C*_1_>*C*_2_>⋯>*C*_*p*_ is satisfied and *p*_0_ is satisfied by Eq (7), then the assessment object *X*_*i*_ belongs to the assessment grade $C_{p_{0}}$.

$$p_{0}=\min\left|p:\sum_{k=1}^{p}\mu_{ik}>\lambda ,\;i=1,2,\cdots ,\;n\right|$$ (7)

### Determination of the index weight

The entropy weight method [46, 47] is used to determine the weight of each index, and this method can make full use of the values of the single-index unascertained measure matrix.

Suppose *w*_*j*_ denotes the relative degree of importance of an index compared with other indexes. If *w*_*j*_ satisfies 0≤*w*_*j*_≤1 and $\sum_{j=1}^{m}w_{j}=1$, then *w*_*j*_ is called the index weight of *V*_*j*_, and *w* = (*w*_1_,*w*_2_,⋯,*w*_*m*_) is called the vector of the index weight. According to the matrix (*μ*_*ijk*_)_*m*×*p*_, the index weight *w*_*j*_ can be obtained from Eqs (8) and (9).

$$H_{j}=-t\sum_{k=1}^{p}q_{ijk}\ln q_{ijk}$$ (8)

$$w_{j}=\frac{d_{j}}{\sum_{j=1}^{m}d_{j}}=\frac{1-H_{j}}{m-\sum_{j=1}^{m}H_{j}}$$ (9)

where *H*_*j*_>0; $q_{ijk}=\mu_{ijk}/\sum_{k=1}^{p}\mu_{ijk}$; *t* is a coefficient and *t* = 1/ln *p*; and when *μ*_*ijk*_ = 0, *μ*_*ijk*_ ln *μ*_*ijk*_ = 0 (*i* = 1,2,⋯,*n*).

## Case study

To verify the effectiveness of the model proposed herein, a high-rise building under construction is taken as an example. The building, which is an inpatient building in a hospital in Xi’ an, China, has a shear wall structure with a length of approximately 116 m, a width of approximately 53.7 m and a height of approximately 81.8 m. The building has 21 floors, 2 of which are underground and 19 of which are above ground. The total floor area is 77,260 m^2^, the underground floor area is 10,600 m^2^, and the ground floor area is 66,660 m^2^.

### Data collection

This paper used the expert scoring method to determine the actual assessment value of each index. Ten experts in related fields were invited to inspect the construction site, the basic information of these ten experts is shown in Table 3.

**Table 3:** The basic information of the experts.

Expert code	Professional title	Academic qualification	Working years
E_1_	Senior title	Specialty	25
E_2_	Intermediate title	Undergraduate	15
E_3_	Senior title	Undergraduate	18
E_4_	Intermediate title	Master	8
E_5_	Intermediate title	Undergraduate	12
E_6_	Senior title	Master	21
E_7_	Intermediate title	Undergraduate	14
E_8_	Intermediate title	Master	9
E_9_	Senior title	Master	17
E_10_	Intermediate title	Undergraduate	15

And the third-level indexes were scored by these ten experts according to the index grading standards in Table 2. The specific scores are shown in Table 4. To ensure the objectivity and authenticity of the scores, the highest and lowest scores of the indexes are first eliminated, and then the average value of each index was obtained. The fire risk assessment index value of the project is shown in Table 4.

**Table 4:** The fire risk assessment index value of the project.

Index	Scoring by ten experts										Final index value
	E_1_	E_2_	E_3_	E_4_	E_5_	E_6_	E_7_	E_8_	E_9_	E_10_
V_111_	85	83	87	84	87	83	84	82	84	83	84.13
V_112_	86	88	83	84	85	86	84	84	85	87	85.13
V_113_	87	89	92	90	88	90	88	89	87	89	88.75
V_114_	65	63	64	68	63	64	65	64	66	63	64.25
V_121_	63	65	67	66	68	65	67	66	63	66	65.63
V_211_	92	85	87	90	88	86	91	87	89	86	88.00
V_212_	56	60	58	61	63	58	59	58	61	56	58.88
V_213_	88	83	86	85	87	88	85	87	85	84	85.88
V_221_	89	91	88	85	86	87	90	86	90	87	87.88
V_222_	88	90	93	87	92	87	91	92	91	88	89.88
V_311_	84	87	86	83	82	83	85	84	85	82	84.00
V_312_	85	88	87	84	83	89	83	87	86	83	85.38
V_313_	88	83	85	84	89	83	88	85	88	84	85.63
V_321_	90	88	85	91	89	89	90	89	87	85	88.38
V_322_	87	83	84	86	87	85	84	86	83	87	85.25
V_331_	83	85	79	80	83	82	84	81	85	84	82.75
V_332_	88	90	86	87	83	87	88	85	89	86	87.00
V_341_	89	92	94	90	91	91	90	90	93	91	91.00
V_342_	83	85	86	83	82	86	86	86	82	86	84.63
V_343_	87	89	84	86	87	88	87	86	84	87	86.50
V_411_	84	80	83	81	85	82	84	82	83	81	82.50
V_412_	81	81	79	82	80	81	79	82	82	81	80.88
V_421_	83	81	84	81	82	81	80	84	80	81	81.63
V_422_	83	81	85	81	84	83	81	84	83	83	82.75
V_423_	85	83	87	83	85	85	87	86	84	86	85.13
V_424_	85	86	84	88	84	87	85	84	87	85	85.38
V_431_	87	85	89	85	86	85	85	85	88	85	85.75
V_432_	89	87	88	84	82	86	87	86	85	85	86.00
V_511_	84	88	87	83	82	87	85	82	86	84	84.75
V_512_	85	82	81	83	80	82	81	80	82	84	81.88
V_512_	85	87	84	83	88	83	86	83	84	85	84.63
V_521_	86	87	85	88	83	84	87	87	87	85	86.00
V_522_	80	81	80	84	85	82	80	80	84	80	81.38

### Calculation process

(1) Constructing the single-index unascertained measure function

The unascertained measure function studied in this paper is linear. The single-index unascertained measure function is constructed as follows:

$$\mu_{x\in C_{1}}=\{\begin{array}{cc} 0 & x\leq 85\\ \frac{x-85}{5} & 85<x\leq 90\\ 1 & x>90 \end{array}$$ (10)

$$\mu_{x\in C_{2}}=\{\begin{array}{cc} 0 & x\leq 75\;\mathrm{or}\;x>90\\ \frac{x-75}{10} & 75<x\leq 85\\ \frac{90-x}{5} & 85<x\leq 90 \end{array}$$ (11)

$$\mu_{x\in C_{3}}=\{\begin{array}{cc} 0 & x\leq 65\;\mathrm{or}\;x>85\\ \frac{x-65}{10} & 65<x\leq 75\\ \frac{85-x}{10} & 75<x\leq 85 \end{array}$$ (12)

$$\mu_{x\in C_{4}}=\{\begin{array}{cc} 0 & x\leq 60\;\mathrm{or}\;x>75\\ \frac{x-60}{5} & 60<x\leq 65\\ \frac{75-x}{10} & 65<x\leq 75 \end{array}$$ (13)

$$\mu_{x\in C_{5}}=\{\begin{array}{cc} 1 & x\leq 60\\ \frac{65-x}{5} & 60<x\leq 65\\ 0 & x>65 \end{array}$$ (14)

**Fig 2:**
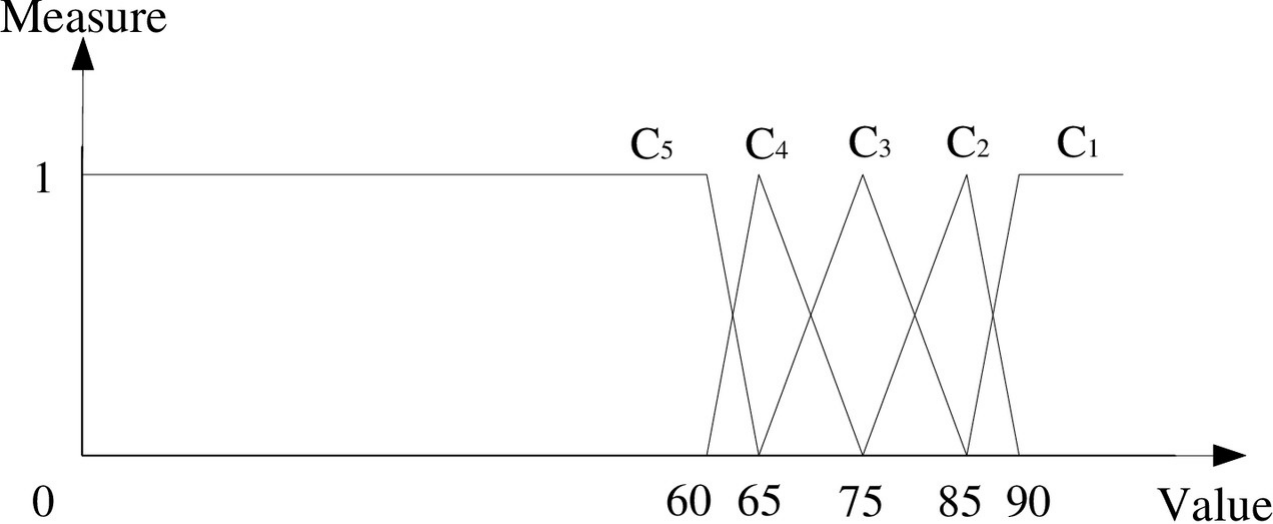
The single-index unascertained measure function.

In this paper, the single-index unascertained measure function is represented by a graph, as shown in Fig 2.

(2) Calculating the single-index unascertained measure matrix

According to the index value in Table 4 and based on the single-index unascertained measure function established by Eq (10)–Eq (14), the three-level index unascertained measure matrix can be obtained.

$$\mu_{11}=\left[\begin{array}{ccccc} 0.000 & 0.913 & 0.088 & 0.000 & 0.000\\ 0.025 & 0.975 & 0.000 & 0.000 & 0.000\\ 0.750 & 0.250 & 0.000 & 0.000 & 0.000\\ 0.000 & 0.000 & 0.000 & 0.850 & 0.150 \end{array}\right]\;\mu_{12}=\left[\begin{array}{ccccc} 0.000 & 0.000 & 0.063 & 0.938 & 0.000 \end{array}\right]$$

$$\mu_{21}=\left[\begin{array}{ccccc} 0.600 & 0.400 & 0.000 & 0.000 & 0.000\\ 0.000 & 0.000 & 0.000 & 0.000 & 1.000\\ 0.175 & 0.825 & 0.000 & 0.000 & 0.000 \end{array}\right]\;\mu_{22}=\left[\begin{array}{ccccc} 0.575 & 0.425 & 0.000 & 0.000 & 0.000\\ 0.975 & 0.025 & 0.000 & 0.000 & 0.000 \end{array}\right]$$

$$\mu_{31}=\left[\begin{array}{ccccc} 0.000 & 0.900 & 0.100 & 0.000 & 0.000\\ 0.075 & 0.925 & 0.000 & 0.000 & 0.000\\ 0.125 & 0.875 & 0.000 & 0.000 & 0.000 \end{array}\right]\;\mu_{32}=\left[\begin{array}{ccccc} 0.675 & 0.325 & 0.000 & 0.000 & 0.000\\ 0.050 & 0.950 & 0.000 & 0.000 & 0.000 \end{array}\right]$$

$$\mu_{33}=\left[\begin{array}{ccccc} 0.000 & 0.775 & 0.225 & 0.000 & 0.000\\ 0.400 & 0.600 & 0.000 & 0.000 & 0.000 \end{array}\right]\;\mu_{34}=\left[\begin{array}{ccccc} 1.000 & 0.000 & 0.000 & 0.000 & 0.000\\ 0.000 & 0.963 & 0.038 & 0.000 & 0.000\\ 0.300 & 0.700 & 0.000 & 0.000 & 0.000 \end{array}\right]$$

$$\mu_{41}=\left[\begin{array}{ccccc} 0.000 & 0.750 & 0.250 & 0.000 & 0.000\\ 0.000 & 0.588 & 0.413 & 0.000 & 0.000 \end{array}\right]\;\mu_{42}=\left[\begin{array}{ccccc} 0.000 & 0.663 & 0.338 & 0.000 & 0.000\\ 0.000 & 0.775 & 0.225 & 0.000 & 0.000\\ 0.025 & 0.975 & 0.000 & 0.000 & 0.000\\ 0.075 & 0.925 & 0.000 & 0.000 & 0.000 \end{array}\right]$$

$$\mu_{43}=\left[\begin{array}{ccccc} 0.150 & 0.850 & 0.000 & 0.000 & 0.000\\ 0.200 & 0.800 & 0.000 & 0.000 & 0.000 \end{array}\right]\;\mu_{51}=\left[\begin{array}{ccccc} 0.000 & 0.975 & 0.025 & 0.000 & 0.000\\ 0.000 & 0.688 & 0.313 & 0.000 & 0.000\\ 0.000 & 0.963 & 0.038 & 0.000 & 0.000 \end{array}\right]$$

$$\mu_{52}=\left[\begin{array}{ccccc} 0.200 & 0.800 & 0.000 & 0.000 & 0.000\\ 0.000 & 0.638 & 0.363 & 0.000 & 0.000 \end{array}\right]$$

(3) Determining the index weight

According to the previous content, the index weight is calculated, as shown in Table 5.

**Table 5:** Index weights.

First-level index	First-level index weight	Second-level index	Second-level index weight	Third-level index	Third-level index weight
V_1_	0.178	V_11_	0.255	V_111_	0.261
				V_112_	0.296
				V_113_	0.208
				V_114_	0.236
		V_12_	0.745	V_121_	1.000
V_2_	0.158	V_21_	0.327	V_211_	0.254
				V_212_	0.436
				V_213_	0.310
		V_22_	0.673	V_221_	0.383
				V_222_	0.617
V_3_	0.206	V_31_	0.317	V_311_	0.333
				V_312_	0.348
				V_313_	0.319
		V_32_	0.257	V_321_	0.410
				V_322_	0.590
		V_33_	0.204	V_331_	0.535
				V_332_	0.465
		V_34_	0.222	V_341_	0.397
				V_342_	0.357
				V_343_	0.246
V_4_	0.218	V_41_	0.301	V_411_	0.529
				V_412_	0.471
		V_42_	0.346	V_421_	0.199
				V_422_	0.220
				V_423_	0.306
				V_424_	0.275
		V_43_	0.353	V_431_	0.517
				V_432_	0.483
V_5_	0.240	V_51_	0.605	V_511_	0.380
				V_512_	0.251
				V_512_	0.369
		V_52_	0.395	V_521_	0.537
				V_522_	0.463

(4) Calculating the comprehensive unascertained measure matrix

1) Calculating the second-level index comprehensive measure matrix

According to the third-level index unascertained measure matrix and the third-level index weight, the second-level index comprehensive measure matrix can be obtained.

$$\mu_{1}=\left[\begin{array}{ccccc} 0.163 & 0.578 & 0.023 & 0.200 & 0.035\\ 0.000 & 0.000 & 0.063 & 0.938 & 0.000 \end{array}\right]\;\mu_{2}=\left[\begin{array}{ccccc} 0.207 & 0.358 & 0.000 & 0.000 & 0.436\\ 0.822 & 0.178 & 0.000 & 0.000 & 0.000 \end{array}\right]$$

$$\mu_{3}=\left[\begin{array}{ccccc} 0.066 & 0.901 & 0.033 & 0.000 & 0.000\\ 0.306 & 0.694 & 0.000 & 0.000 & 0.000\\ 0.186 & 0.694 & 0.120 & 0.000 & 0.000\\ 0.470 & 0.516 & 0.013 & 0.000 & 0.000 \end{array}\right]\;\mu_{4}=\left[\begin{array}{ccccc} 0.000 & 0.673 & 0.327 & 0.000 & 0.000\\ 0.028 & 0.855 & 0.117 & 0.000 & 0.000\\ 0.174 & 0.826 & 0.000 & 0.000 & 0.000 \end{array}\right]$$

$$\mu_{5}=\left[\begin{array}{ccccc} 0.000 & 0.898 & 0.102 & 0.000 & 0.000\\ 0.107 & 0.725 & 0.168 & 0.000 & 0.000 \end{array}\right]$$

2) Calculating the first-level index comprehensive measure matrix

According to the second-level index unascertained measure matrix and the second-level index weight, the first-level index comprehensive measure matrix can be obtained.

$$\mu =\left[\begin{array}{ccccc} 0.042 & 0.147 & 0.052 & 0.750 & 0.009\\ 0.621 & 0.237 & 0.000 & 0.000 & 0.143\\ 0.242 & 0.720 & 0.038 & 0.000 & 0.000\\ 0.071 & 0.790 & 0.139 & 0.000 & 0.000\\ 0.042 & 0.830 & 0.128 & 0.000 & 0.000 \end{array}\right]$$

3) Calculating the total target comprehensive measure matrix

According to the first-level index unascertained measure matrix and the first-level index weight, the total target comprehensive measure matrix can be obtained

$$\mu_{total}=\left[0.181\;0.584\;0.078\;0.133\;0.024\right]$$

(5) Credible degree recognition

The total target comprehensive measure matrix of fire risk of high-rise buildings under construction is *μ*_*total*_ = [0.181 0.584 0.078 0.133 0.024].

According to the obtained measure matrix, the credible degree recognition criterion is used for fire risk assessment. *λ*, which is generally 0.6 or 0.7, is set to 0.6 in this paper. As 0.181+0.584 = 0.765>0.7, *p*_0_ = 2; that is, the fire risk assessment grade of the high-rise building under construction is Grade II and the risk is low, and this assessment result is consistent with the result determined by the fire department. Similarly, the fire risk assessment grades of all indexes can be obtained are shown in Table 6.

**Table 6:** Fire risk assessment grade of each index.

First-level index	Risk grade	Second-level index	Risk grade	Third-level index	Risk grade
V_1_	IV	V_11_	II	V_111_	II
				V_112_	II
				V_113_	I
				V_114_	IV
		V_12_	IV	V_121_	IV
V_2_	II	V_21_	V	V_211_	II
				V_212_	V
				V_213_	II
		V_22_	I	V_221_	II
				V_222_	I
V_3_	II	V_31_	II	V_311_	II
				V_312_	II
				V_313_	II
		V_32_	II	V_321_	II
				V_322_	II
		V_33_	II	V_331_	II
				V_332_	II
		V_34_	II	V_341_	I
				V_342_	II
				V_343_	II
V_4_	II	V_41_	III	V_411_	II
				V_412_	III
		V_42_	II	V_421_	III
				V_422_	II
				V_423_	II
				V_424_	II
		V_43_	II	V_431_	II
				V_432_	II
V_5_	II	V_51_	II	V_511_	II
				V_512_	III
				V_512_	II
		V_52_	II	V_521_	II
				V_522_	III

## Results and discussion

Through the analysis of the above assessment results, it is concluded that the actual fire risk level of the case is Grade II, which represents a low fire risk and an acceptable state. From Table 6, we can see the assessment results of each index level. Among them, the risk level of the “passive fire prevention factor” is Grade IV, while the risk levels of the “active fire prevention factor, the safe evacuation factor, fire safety management and fire hazard control” are Grade II.

After obtaining the fire risk grade of the project, the project leader, management personnel, technical personnel and relevant experts conducted a field investigation, discussed and analyzed the actual situation of the project. For the “passive fire prevention factor”, the project is in the sealing stage of the main body structure, and the building is very tall. Once a fire occurs, the chimney effect and fire load will be very large. In addition, the construction site of the project occupies a relatively small area and piles a lot of materials. There are a large number of people on the site, food and accommodation are all on the construction site, and most of the building materials and workers' daily necessities are flammable, which greatly increases the fire load on the construction site. For the “active fire prevention factor”, the fire protection of the project is relatively in place, but the fire detection and automatic alarm equipment is just like being idle and cannot be used. For the “safe evacuation factor”, the overall situation is good. For the “fire safety management”, the on-site fire fighting facilities are well equipped, a sufficient number of fire extinguishers are installed, and the fire fighting facilities are reasonably distributed, but most of these facilities are idle and unusable. For the “fire hazard control”, there are illegal operations of electrical equipment and unlawful pulling of wires on site.

Although the overall fire risk level of this project is Grade II, as the height of the building increases, it is very important to pay attention to fire prevention in the subsequent construction process. For those indexes with risk level of Grade V and Grade IV, measures must be taken immediately to deal with them, and they must be carefully supervised and inspected.; for those indexes with risk level of Grade III and Grade II, measures should be taken to deal with them according to the specific circumstances; for those indexes with risk level of Grade I, it is not necessary to deal with them.

By analyzing the basic situation of the project and comparing the assessment results of the model with the on-site situation, it is confirmed that the assessment results are scientific, reasonable, and consistent with the on-site situation. Based on the results, the project managers should strengthen the configuration and supervision of the fire extinguishing facilities of the building itself, as well as the fire control management, and adopt reward and punishment measures. In addition, the project managers should strengthen the fire safety investigation at the construction site, monitor the potential fire hazards at all times, and rectify them immediately to minimize the possibility of a fire.

According to the above analysis, the assessment model established in this paper is feasible. It shows that in view of the various uncertainties in the fire risk assessment of high-rise buildings under construction, this method can obtain reasonable assessment results. In addition, the calculation process of the model is simple. In summary, the assessment model can handle the uncertainty of fire risk assessment, and it is very suitable for the fire risk assessment of high-rise buildings under construction.

## Limitations

The unascertained measure theory can be used for the fire risk assessment of high-rise buildings under construction, but the study has several limitations. Due to the project is under construction and the fire hazards are in a state of change in each stage. The index system established in this paper is a static index system. More research is needed to establish a dynamic assessment index system and to determine the fire hazards in each stage from time to time.

## Conclusions

To discover hidden fire hazards and to reduce the occurrence of fire accidents in high-rise building under construction, this paper studied the fire risk assessment of high-rise buildings under construction. First, a fire risk assessment index system for high-rise buildings under construction was established, including 5 first-level indexes, 13 second-level indexes and 33 third-level indexes. Second, according to the uncertainty of the fire influence factors of high-rise buildings under construction and the uncertainty of the assessment process, a fire risk assessment model for high-rise buildings under construction based on unascertained measure theory was proposed. Finally, the feasibility and rationality of the proposed fire risk assessment index system and assessment model were verified by taking an inpatient building project of a hospital in Xi’an as an example. This study can solve the problem of fire risk assessment and provide new ideas and methods for the fire risk assessment and control of high-rise buildings under construction in the future.
